# Interleukin-33 signaling exacerbates experimental infectious colitis by enhancing gut permeability and inhibiting protective Th17 immunity

**DOI:** 10.1038/s41385-021-00386-7

**Published:** 2021-03-02

**Authors:** Vittoria Palmieri, Jana-Fabienne Ebel, Nhi Ngo Thi Phuong, Robert Klopfleisch, Vivian Pham Vu, Alexandra Adamczyk, Julia Zöller, Christian Riedel, Jan Buer, Philippe Krebs, Wiebke Hansen, Eva Pastille, Astrid M. Westendorf

**Affiliations:** 1 Institute of Medical Microbiology, University Hospital Essen, University of Duisburg-Essen, Essen, Germany; 2 Institute of Veterinary Pathology, Free University of Berlin, Berlin, Germany; 3 Institute of Pathology, University of Bern, Bern, Switzerland; 4 Graduate School for Cellular and Biomedical Sciences, University of Bern, Bern, Switzerland; 5 Institute of Microbiology and Biotechnology, University of Ulm, Ulm, Germany

## Abstract

A wide range of microbial pathogens is capable of entering the gastrointestinal tract, causing infectious diarrhea and colitis. A finely tuned balance between different cytokines is necessary to eradicate the microbial threat and to avoid infection complications. The current study identified IL-33 as a critical regulator of the immune response to the enteric pathogen *Citrobacter rodentium*. We observed that deficiency of the IL-33 signaling pathway attenuates bacterial-induced colitis. Conversely, boosting this pathway strongly aggravates the inflammatory response and makes the mice prone to systemic infection. Mechanistically, IL-33 mediates its detrimental effect by enhancing gut permeability and by limiting the induction of protective T helper 17 cells at the site of infection, thus impairing host defense mechanisms against the enteric pathogen. Importantly, IL-33-treated infected mice supplemented with IL-17A are able to resist the otherwise strong systemic spreading of the pathogen. These findings reveal a novel IL-33/IL-17A crosstalk that controls the pathogenesis of *Citrobacter rodentium*-driven infectious colitis. Manipulating the dynamics of cytokines may offer new therapeutic strategies to treat specific intestinal infections.

## Introduction

Infectious diarrhea and colitis due to pathogenic intestinal bacteria represent a major health problem in both developing and developed countries^1^. Consequently, a thorough understanding of the infectious process and the underlying immunological responses is required for the identification and treatment of these infectious diseases. *Citrobacter rodentium* (CR) is a non-invasive attaching/effacing enteric pathogen, which infects intestinal epithelial cells (IECs) and causes infectious colitis in mice^2^. Thus, it is routinely used to mimic human pathogenic *Escherichia coli* infections and to study host-pathogen interactions. Similar to other enteropathogens, CR elicits a strong Th1 and Th17 immune response in the colon, which is important for the clearance of infection^3^. Th1 cells produce interferon-γ (IFN-γ) and tumor necrosis factor-α (TNF-α), promoting macrophage and cytotoxic T cell activation. Th17 cells are induced by IL-1β, IL-6, transforming growth factor-β, and release IL-17A, IL-17F, and IL-22, which are crucial for the host defense and bacterial clearance by inducing the production of antimicrobial peptides and fostering the maintenance of intestinal barrier functions^4^. While an appropriate cytokine release is required to mount an effective immune response against microbial pathogens, excessive production of some cytokines can sustain the inflammatory process and increase the risk of infection complications.

IL-33, a member of the IL-1 cytokine family^5^, has become of great interest in the field of intestinal inflammation. IL-33 is constitutively expressed at high levels in epithelial barrier tissues such as the intestine, and is primarily secreted by fibroblasts, epithelial cells, and endothelial cells in response to injury^6^. Upon release, IL-33 binds to the receptor ST2, which exists in two forms as splice variants: a soluble form (sST2), which acts as a decoy receptor, sequesters free IL-33, and does not signal, and a membrane-bound form (ST2), which activates the MyD88/NF-κB signaling pathway to modulate immune cell functions^7^. Several immune cells, from both the innate and adaptive lineages, are targets of IL-33 as they express the membrane-bound receptor ST2 either constitutively or transiently^8^. Besides immune cells, IL-33 was also shown to target intestinal epithelial progenitors as these cells functionally express ST2^9^. Depending on the physiological context, IL-33 has been described to be host-protective or -pathogenic in the gut^10^. Studies using colitis mouse models have shown that administration of recombinant IL-33 ameliorates intestinal inflammation^11,12^. Furthermore, IL-33^−/−^ mice showed higher susceptibility to infection and severe gut tissue destruction in a model of Salmonellosis^9^. In contrast, during chronic colitis IL-33 was recognized to be detrimental by favoring a tumor-promoting immune environment^13^. Taken together, the precise role of the IL-33/ST2 signaling pathway in the intestine is still ambiguous and must be carefully analyzed with respect to the inflammatory state, the infectious agent, and the specific host immune response involved.

In the present study, we identified that deficiency of the IL-33/ST2 signaling pathway attenuates CR-associated colon pathology. Conversely, boosting this pathway strongly aggravates bacteria-driven colitis and contributes to systemic pathogen dissemination by mitigating the intestinal epithelial integrity. In addition, we identified IL-33 as a negative regulator of Th17 cell differentiation, cells that are essential for the clearance of the pathogen. In summary, our findings reveal the need of a tightly regulated balance between IL-17A and IL-33-mediated signals for the clearance of specific enteric bacterial pathogens and the control of infection-driven intestinal immunopathology.

## Results

### Enhanced colonic ST2 expression following *C. rodentium* infection

**Fig. 1:**
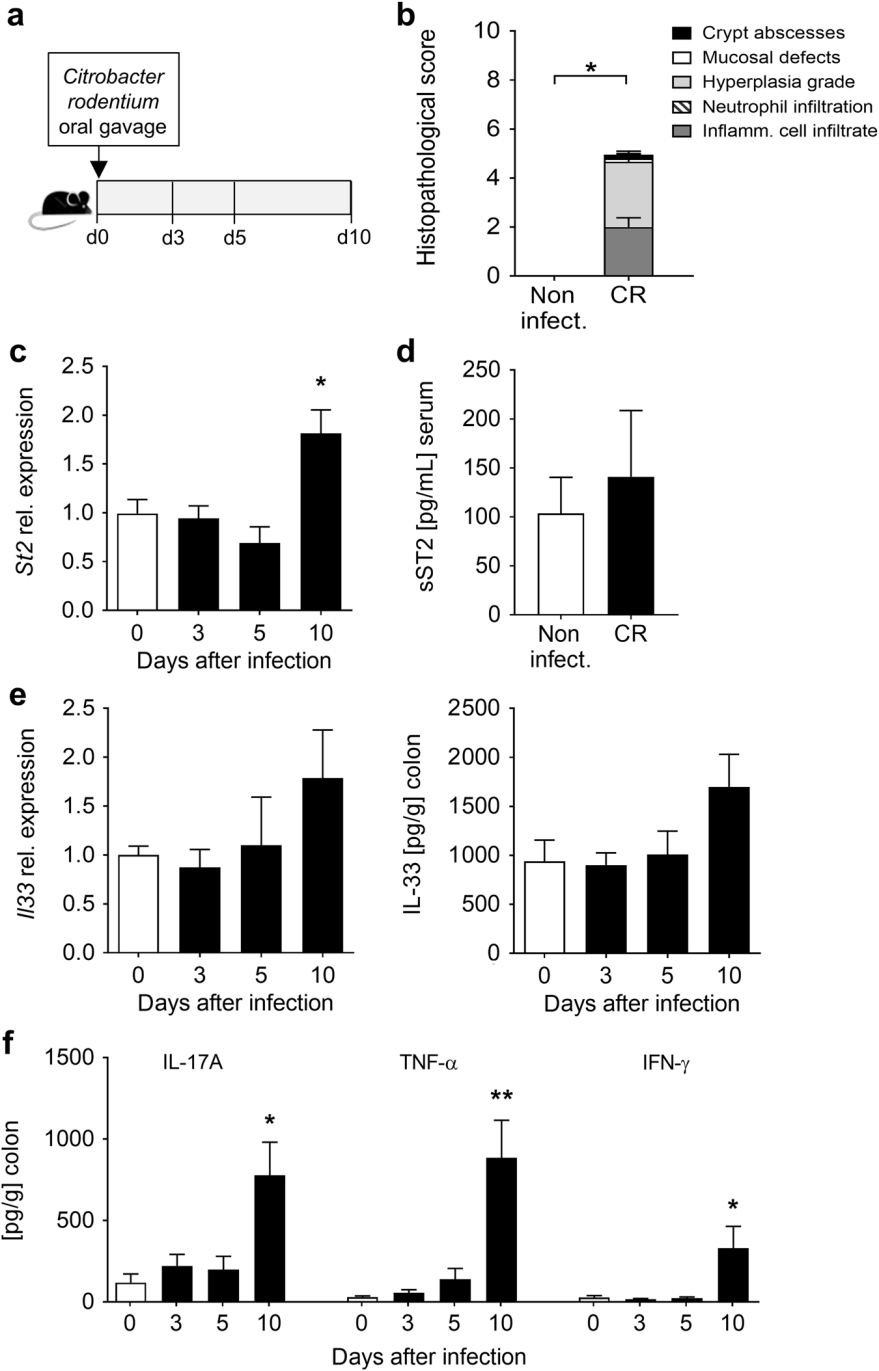
Influence of *C. rodentium* infection on colonic ST2 and IL-33 expression and IL-33 production. **a** C57BL/6 mice were orally gavaged with CR on day 0. **b** Histopathological score of colon tissues assessed 10 days post infection. Bars represent the mean ± SEM of data from one representative experiment (*n* = 3 mice per group). **c** Colonic mRNA expression of the membrane-bound receptor ST2 measured at the indicated time points after infection by qRT-PCR. Bars represent fold change induction over d0 (*n* = 5–11 mice per group). **d** Serum sST2 concentrations determined on day 10 after infection via ELISA. **e** *Il33* transcript levels (left panel) in colon biopsies and IL-33 protein secretion from in vitro cultured colon explants (right panel) were measured via RT-PCR and Luminex technology, respectively (*n* = 5–6 mice per group). **f** Secretion of IL-17A, TNF-α and IFN-γ from in vitro cultured colon explants determined by Luminex technology (*n* = 5–6 mice per group). All data are presented as mean ± SEM and were pooled from (**c**) three or (**d**, **e**, **f**) two independent experiments. Statistical analyses were performed using Mann–Whitney U test or Student’s *t* test. *P < 0.05; **P < 0.01; ***P < 0.001.

IL-33 and its transmembrane receptor ST2 have been described as important players in the maintenance of gut homeostasis^14^. To determine whether the expression of ST2 and IL-33 is altered during CR infection, wild-type (WT) C57BL/6 mice were infected with CR and analyzed at different time points after infection (Fig. 1a). Bacterial-induced colitis was confirmed on day 10 after infection by colonic histopathological analysis (Fig. 1b). Remarkably, we detected a significant upregulation of the membrane-bound isoform of ST2 (*St2*) at mRNA levels in the colons of infected mice compared to non-infected animals on day 10 after infection (Fig. 1c), but no significant alterations in the soluble form (sST2) in the serum (Fig. 1d). Interestingly, IL-33 was highly abundant in the colon under steady-state, and *Il33* transcripts and IL-33 protein were slightly but not significantly increased at day 10 post infection (Fig. 1e). Conversely, IL-17A, TNF-α, and IFN-γ secretion were strongly induced in the colon of CR-infected mice compared to non-infected mice at day 10 of infection (Fig. 1f). Taken together, these data suggest that IL-33/ST2 signaling is controlled by the expression of the transmembrane receptor ST2 rather than via the modulation of IL-33 levels during enteric infection with CR.

### Lack of IL-33/ST2 signaling ameliorates *C. rodentium*-induced colitis

**Fig. 2:**
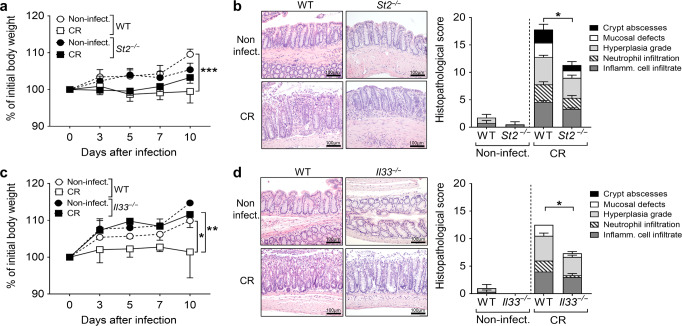
Lack of IL-33 or ST2 alleviates *C. rodentium*-induced intestinal inflammation. **a** Body weight changes relative to initial weight in *St2*^*−/−*^ and WT non-infected and CR-infected mice monitored during the course of the experiment. Statistical analyses were performed using two-way ANOVA followed by Bonferroni’s post hoc test. **b** Representative pictures (scale bars 100 µm) of H&E-stained colon sections (left) and histopathological score (right). Each parameter of colon damage and inflammation is shown as mean ± SEM. Statistical analyses were performed using Mann–Whitney U test or Student’s *t* test to compare *St2*^*−/−*^ groups with their respective WT control groups. Results from two independent experiments are shown (*n* = 4–6 per group). **c** Body weight changes relative to initial weight in *Il33*^*−/−*^ and WT non-infected and CR-infected mice. Statistical analysis was performed using two-way ANOVA followed by Bonferroni’s post hoc test. **d** Representative pictures (scale bars 100 µm) of H&E-stained colon sections (left) and histopathological score (right). Middle and distal colon portions were assessed for damage and inflammation as before. Bars show the mean ± SEM of each parameter. Statistical analysis was performed using Mann–Whitney U test or Student’s *t* test to compare *Il33*^*−/−*^ groups with their respective WT control groups (*n* = 3 mice per group). *P < 0.05; **P < 0.01; ***P < 0.001.

To address the physiological function of IL-33/ST2 signaling during intestinal bacterial infection, we first infected wild-type controls (WT) and ST2 knockout mice (*St2*^*−/−*^) as well as IL-33 knockout mice (*Il33*^*−/−*^) with CR for 10 days. Whereas CR-infected WT mice showed reduced body weight in comparison to non-infected WT controls at day 10, no differences in the body weight were observed between infected and non-infected knockout mice (Fig. 2a and c). Well in line, colon damage and inflammation (histopathological score) were significantly reduced in CR-infected *St2*^*−/−*^ compared to WT mice (Fig. 2b). Likewise, *Il33*^−/−^ mice displayed less pathological alterations in the infected colon (Fig. 2d). Collectively, these data provide evidence that the IL-33/ST2 axis contributes to CR-induced pathogenesis.

### IL-33 administration aggravates *C. rodentium* infection

**Fig. 3:**
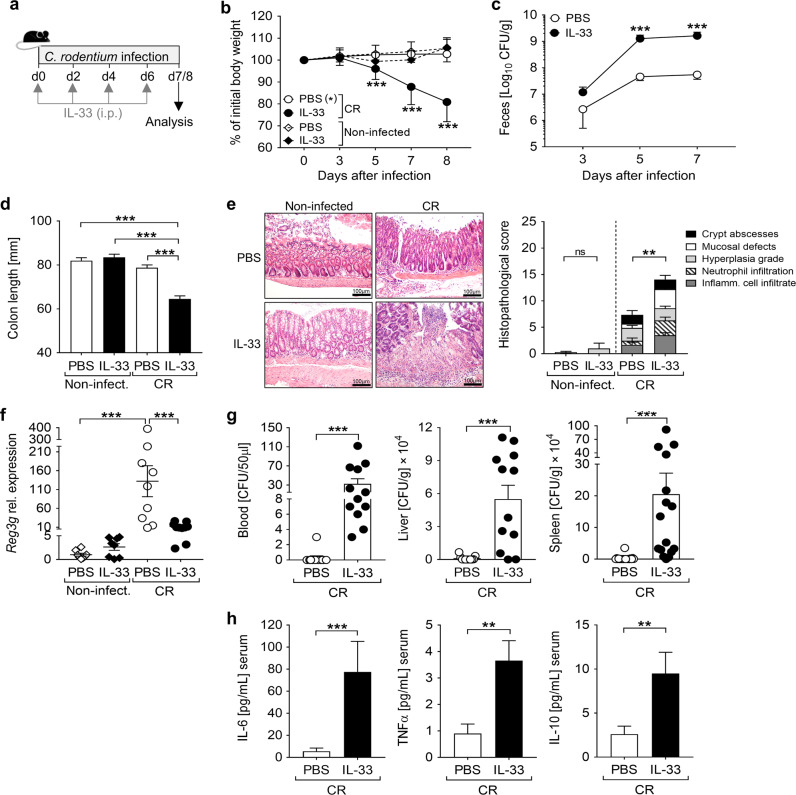
Detrimental effect of IL-33 administration during *C. rodentium* infection. **a** C57BL/6 mice were orally gavaged with CR on day 0. Non-infected and CR-infected mice received IL-33 or PBS (control groups) intraperitoneally on day 0, 2, 4, and 6 after infection. Mice were sacrificed before the body weight loss exceeded 20%. **b** Body weight was monitored daily during infection (*n* = 18–23 mice per group). Statistical analysis was performed using two-way ANOVA followed by Bonferroni’s post hoc test. **c** Bacterial load in feces assessed at the indicated time points after infection (*n* = 22–23 mice per group). Statistical analyses were performed using Mann–Whitney U test to compare groups at each time point. **d** Macroscopic score of colitis based on shortening of colon length (*n* = 18–23 mice per group). One-way ANOVA followed by Tukey’s multiple comparison test was used for statistical analysis. All data are presented as mean ± SEM and were pooled from seven independent experiments. **e** Representative pictures (scale bars 100 µm) of H&E-stained colon sections (left) and histopathological score (right). Bars show the mean ± SEM of each inflammatory parameter analyzed. Data from three independent experiments are shown (*n* = 8–10 mice per group). Statistical analyses were performed using Student’s *t* test to compare IL-33-treated groups with non-treated groups, respectively with or without infection. **f** Colonic *Reg3g* mRNA expression determined by qRT-PCR. Bars show the mean ± SEM of fold change induction over non-infected, non-treated group. One-way ANOVA followed by Tukey’s multiple comparison test was used for statistical analysis. Results from three experiments are shown. **g** Systemic bacterial distribution was assessed by plating whole blood and serial dilutions of homogenized tissues on MacConkey agar. Bars represent the mean ± SEM of data from three experiments. **h** In the same experiments, serum levels of inflammatory cytokines were measured via Luminex technology. Bars indicate the mean ± SEM of cytokine picograms per milliliters of serum (*n* = 11–12 mice per group). Statistical analysis was performed using Mann–Whitney U test. *P < 0.05; **P < 0.01; ***P < 0.001.

Next, we assessed whether the host response to CR infection is altered by further boosting ST2 signaling. Thus, CR-infected and naïve mice were treated with IL-33 on the day of infection and every second day for the duration of the experiment (Fig. 3a). IL-33-treated CR-infected mice showed a dramatic loss of body weight from day 5 on compared to PBS-treated infected mice, with up to 20% reduction at day 8 of infection (Fig. 3b). These mice also suffered from severe diarrhea, mild rectal bleeding and strongly enhanced intestinal CR burden compared to PBS-treated CR-infected controls (Fig. 3c). Moreover, IL-33-treated infected animals showed shorter colons (Fig. 3d) and more severe colon pathology with extensive mucosal defects and inflammatory cell infiltration into the lamina propria in comparison to infected controls (Fig. 3e). Importantly, we observed no signs of intestinal inflammation when IL-33 was administered to non-infected mice. Nevertheless, in accordance with previous studies^15^, colon tissues from non-infected mice subjected to IL-33 treatment revealed mild hyperplasia (Fig. 3e). Given the enhanced CR burden in the feces of IL-33-treated infected mice, we checked whether IL-33 treatment interferes with the production of antimicrobial peptides that have been shown to contribute to the clearance of CR^16^. Reg3γ expression was strongly induced in the colon of CR-infected mice in comparison to non-infected controls. In contrast, mice treated with IL-33 during infection showed a substantial reduction in Reg3γ expression (Fig. 3f). CR infection is normally restricted to the distal colon in C57BL/6 WT mice^17^. Interestingly, we observed strongly enhanced bacterial loads in the blood, liver and spleen of IL-33-treated CR-infected mice but not in PBS-treated infected animals (Fig. 3g), indicating bacterial translocation to systemic compartments. Moreover, serum levels of IL-6, TNFα and IL-10 were strongly increased in CR-infected animals that had received IL-33 (Fig. 3h). Collectively, these findings suggest that IL-33 promotes the intestinal immunopathology associated with CR and the systemic dissemination of the enteric pathogen.

### ST2 is mainly expressed by colonic Tregs during *C. rodentium* infection

**Fig. 4:**
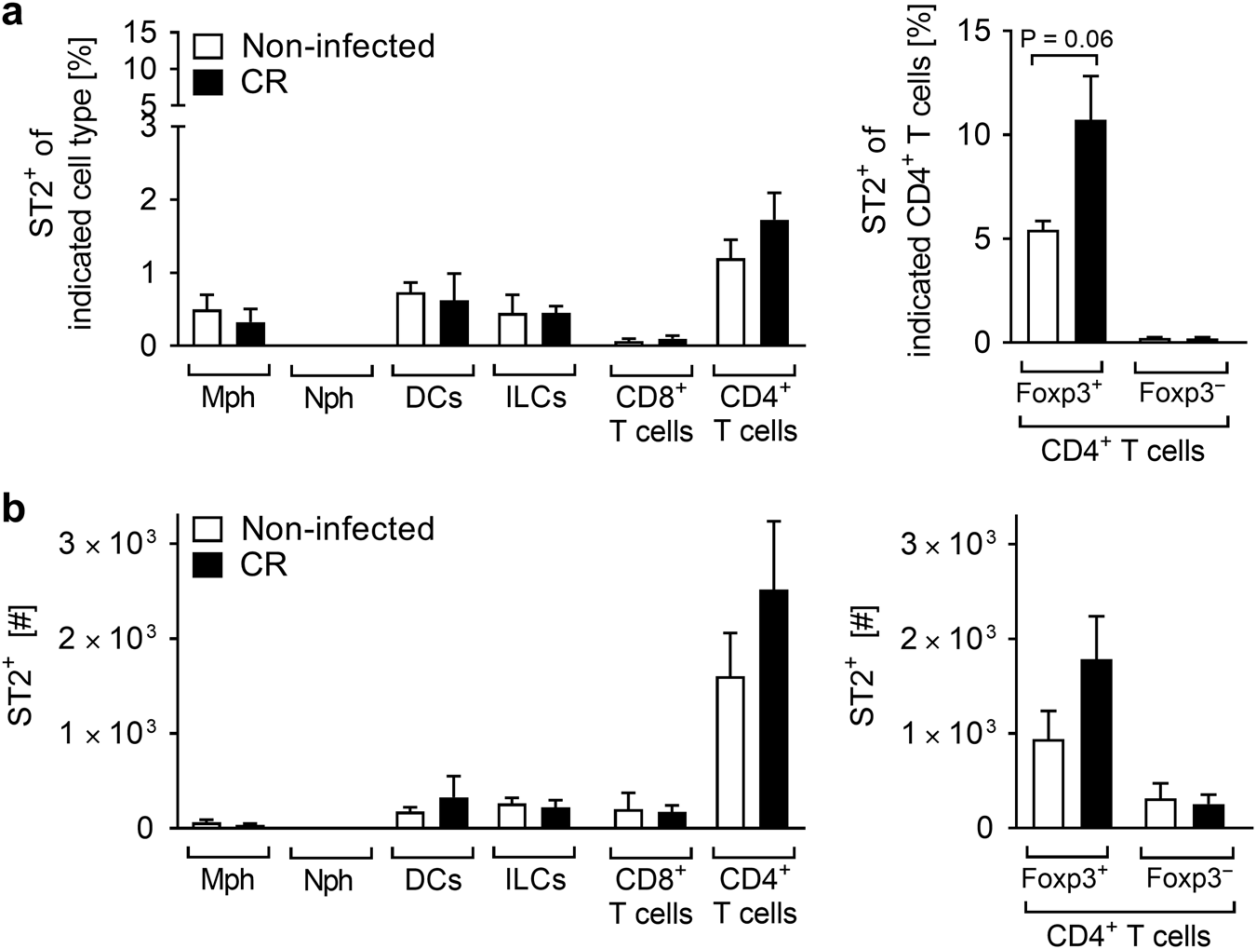
ST2-expressing Treg cells preferentially infiltrate the colonic lamina propria in response to *C. rodentium* infection. Immune cell subsets isolated from the colonic lamina propria of non-infected and CR-infected mice were analyzed by flow cytometry. Frequencies (**a**) and absolute numbers (**b**) of ST2^+^ cells were measured among F4/80^+^CD11b^+^ macrophages (Mph), Ly6G^+^CD11b^+^ neutrophils (Nph), CD11c^+^CD11b^-^F4/80^-^ dendritic cells (DCs), Lin^-^CD127^+^ innate lymphoid cells (ILCs), CD8^+^ T cells, CD4^+^ T cells, and among Foxp3^+^CD4^+^ T cells (Tregs) and Foxp3^-^CD4^+^ T cells. Data from one experiment are shown as mean ± SEM (*n* = 3-4 mice per group). Statistical analyses were performed using Mann–Whitney U test to compare each infected group with the respective non-infected control group. *P < 0.05; **P < 0.01; ***P < 0.001.

IL-33 triggers multiple functions in several ST2-expressing cells^14^. To identify the immune cells that potentially react to IL-33 to cause the severe phenotype observed in our model, we next determined which ST2^+^ immune cells changed in frequency and number upon CR infection. ST2^+^ macrophages, DCs and ILCs were found at very low percentages and numbers in the colonic lamina propria at steady-state and during CR infection. Among T lymphocytes, CD4^+^ T cells represented the highest proportion of ST2^+^ cells, with a tendency to increase in the colon of CR-infected mice, which was in particular the case for Foxp3^+^CD4^+^ regulatory T cells (Tregs) (Fig. 4a, b). Thus, we first focused our analysis on this regulatory T cell subset.

### The detrimental effect of IL-33 is independent of ST2-expressing Treg expansion

**Fig. 5:**
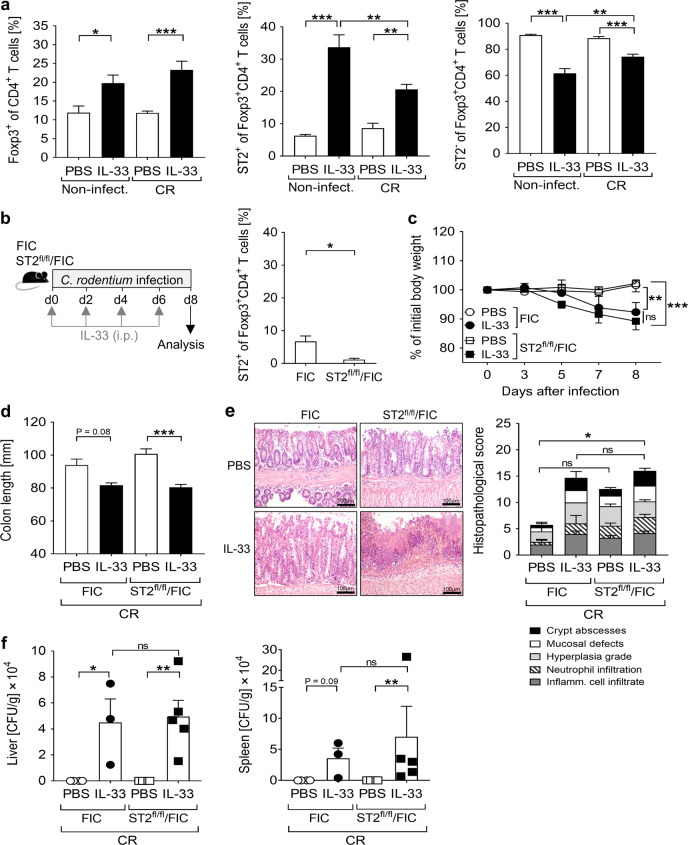
Effect of Treg-specific ST2 ablation on *C. rodentium*-induced colitis upon IL-33 treatment. **a** Mice were treated as described in Fig. 3a. Colonic frequencies of Foxp3^+^ cells (Tregs) among CD4^+^ T cells (left) and of ST2^+^ (middle) or ST2^−^ cells (right) among Tregs determined by flow cytometry. Bars indicate the mean±SEM of data from two independent experiments (*n* = 6–7 mice per group). Statistics were performed using one-way ANOVA followed by Tukey’s multiple comparison test. **b** Schematic illustration of IL-33 treatment during CR infection in ST2^fl/fl^/FIC transgenic mice and control FIC littermates. Colonic Treg-specific ST2 ablation was confirmed via flow cytometry. Each bar shows the mean±SEM of 4 mice. Statistical significances were calculated using Mann–Whitney U test. **c** Body weight changes represented as percentage of initial weight on day 0. Statistical analysis was performed using two-way ANOVA followed by Bonferroni’s post hoc test. **d** Macroscopic score of colitis based on colon length shortening. **e** Representative pictures (scale bars 100 µm) of H&E-stained colon sections (left) and histopathological score (right). Bars show the mean ± SEM of each parameter analyzed. **f** Systemic bacterial distribution assessed by plating serial dilutions of homogenized livers and spleens on MacConkey agar. Dot plots and the mean ± SEM of CFU per grams of tissue are shown. Statistical analysis was performed using one-way ANOVA followed by Tukey’s multiple comparison test or Kruskal-Wallis ANOVA with Dunn’s multiple comparison test. All the data shown derive from two independent experiments. *P < 0.05; **P < 0.01; ***P < 0.001.

IL-33 has been described to expand Tregs and sustain their suppressive functions under inflammatory conditions^18^. Moreover, enhanced Treg frequencies are often associated with reduced T cell effector function during infection, which can lead to diminished pathogen clearance and severe pathogen-induced inflammation^19^. In agreement with these previous reports, IL-33 treatment increased the frequencies of CD4^+^Foxp3^+^ Tregs and particularly of ST2^+^CD4^+^Foxp3^+^ Tregs in both CR-infected and non-infected mice (Fig. 5a and Supplementary Fig. 1a). To determine whether the increased frequencies of ST2^+^ Tregs in IL-33-treated animals accounted for the severe course of disease in CR-infected mice, we performed infection experiments with and without IL-33 treatment of ST2^flox/flox^ x FIC-Cre mice (ST2^fl/fl^/FIC), in which ST2 is ablated specifically in Foxp3^+^ Tregs (Fig. 5b). Surprisingly, no significant differences were observed in body weight loss, colon length and immunopathology, when either ST2-sufficient (FIC) or ST2-deficient Treg (ST2^fl/fl^/FIC) infected animals were treated with IL-33 (Fig. 5c–e). More importantly, both groups showed comparable systemic bacterial dissemination upon IL-33 application (Fig. 5f). Thus, the specific accumulation of ST2-expressing Tregs induced by IL-33 is not responsible for the uncontrolled pathogen dissemination and severe disease progression during CR infection. To determine the overall impact of Tregs on the outcome of CR infection in the presence of IL-33, we next used DEREG mice in our model, which allow the specific depletion of all Foxp3^+^ Tregs by diphtheria toxin application (Supplementary Fig. 1b). In line with the phenotype seen in ST2^fl/fl^/FIC mice, both Treg-sufficient and Treg-depleted CR-infected mice treated with IL-33 showed similar high susceptibility to CR infection (Supplementary Fig. 1c–e). In summary, Tregs seem not to drive the enhanced pathology induced by IL-33 during CR infection. Consequently, other cell types targeted by IL-33 must be responsible for the harmful course of CR-driven colitis.

### IL-33 dampens IL-17A expression during *C. rodentium* infection and interferes with Th17 cell differentiation

**Fig. 6:**
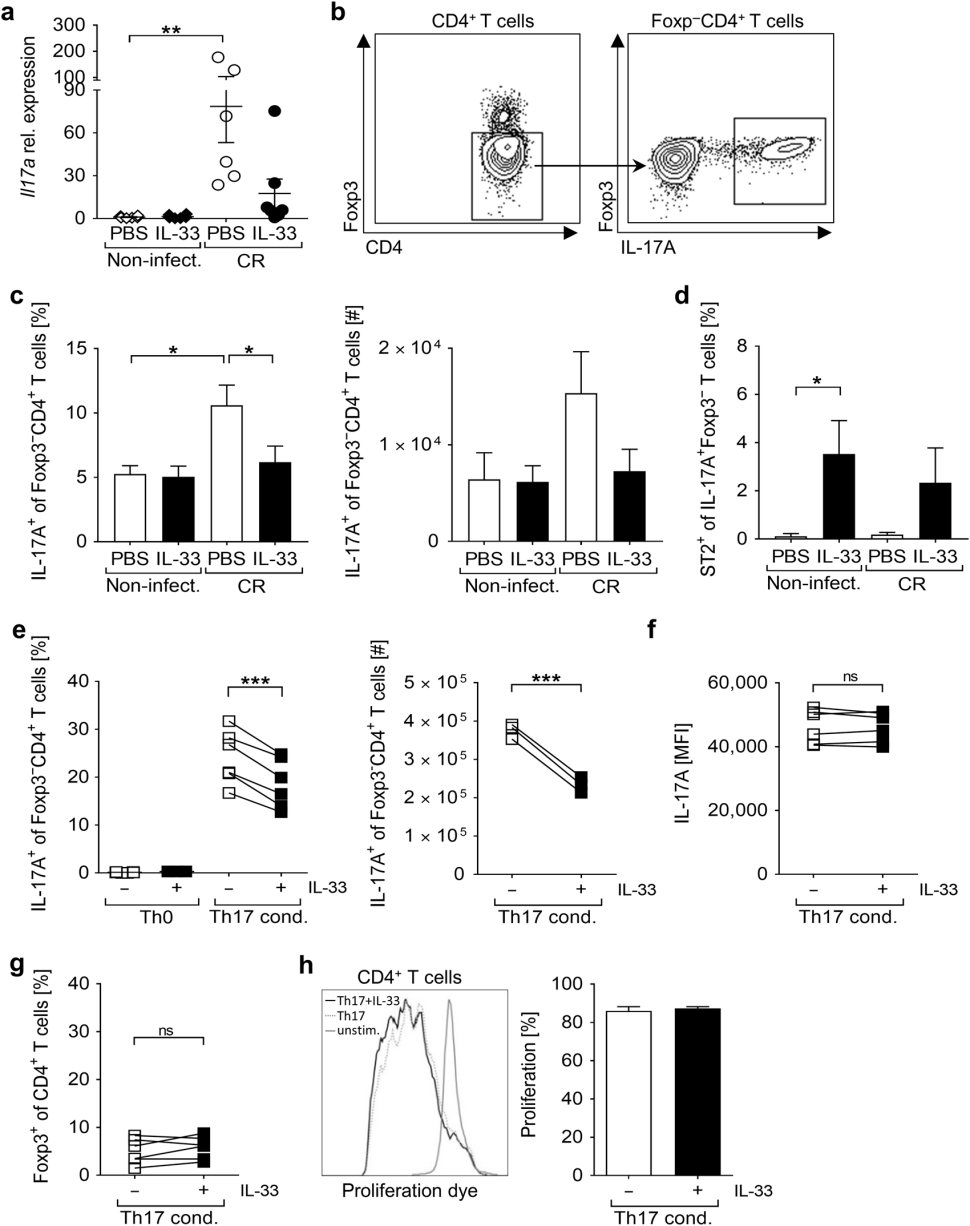
IL-33 interferes with the differentiation of IL-17A-producing Th17 cells. Mice were treated as described in Fig. 3a. **a** qRT-PCR analysis of *Il17a* mRNA expression in colon biopsies from non-infected and CR-infected WT mice treated or not with IL-33. Data from two independent experiments are shown as mean ± SEM of fold change induction over non-infected, PBS-treated group. **b** Flow cytometry dot plots of one representative infected colon showing the gating strategy applied to assess the proportion of IL-17A^+^ cells among Foxp3^-^CD4^+^ T cells (right panel) within the colonic lamina propria. **c** Frequencies and absolute numbers of IL-17A^+^ cells were measured among Foxp3^-^CD4^+^ T cells. **d** Percentage of ST2^+^ cells among Th17 cells. Bars represent the mean ± SEM of data from three independent experiments (*n* = 9–12 mice per group). All statistical analyses were performed using one-way ANOVA followed by Tukey’s multiple comparison test or Kruskal-Wallis ANOVA with Dunn’s multiple comparison test. **e** Sort-purified splenic CD4^+^CD25^-^ T cells were cultured for 5 days under Th17-polarizing conditions in presence or absence of IL-33. On day 5, cells were counted and stained for FACS analysis. The same gating strategy applied to colonic LPLs and shown in panel b was used in this particular dataset to measure the percentage and absolute numbers of IL-17A-expressing cells among Foxp3^-^CD4 T cells (**e**), and to assess their mean fluorescent intensity (MFI) for IL-17A (**f**). In the same experiments, the percentage of Foxp3^+^ cells among CD4^+^ T cells was evaluated (**g**). Data from two independent experiments are shown (*n* = 6 mice). **h** Sort-purified CD4^+^CD25^-^ T cells were labeled with the proliferation dye eFluor 670 and cultured for 5 days under Th17-polarizing conditions with or without IL-33. Proliferation was determined as loss of eFluor 670 by flow cytometry. Representative histogram-overlay is illustrated. Bars indicate the mean ± SEM of *n* = 2 mice. Statistical analyses were performed using Paired *t* test. *P < 0.05; **P < 0.01; ***P < 0.001.

Intestinal infection with CR requires the accumulation of IL-17A-producing cells to clear the pathogen^20^. Strikingly, IL17A expression was markedly enhanced in CR-infected mice but not in infected mice that were treated with IL-33 (Fig. 6a). To address whether IL-33/ST2 signaling may negatively influence Th17 cell induction within the infected colon, we measured the frequencies of IL-17A^+^Foxp3^-^CD4^+^ T cells in colonic tissues as displayed in Fig. 6b. In agreement with the reduction in IL-17A expression, the frequencies and numbers of Th17 cells were significantly reduced in the colon of IL-33-treated CR-infected mice in comparison with infected controls (Fig. 6c). Colonic Th17 cells barely express ST2 at steady-state, and ST2 expression in these cells was not induced upon CR infection. Notably, we found increased ST2^+^ Th17 cell frequencies in the colon of IL-33-treated naïve mice and, to a lesser extent, during CR infection (Fig. 6d). Interestingly, starting IL-33 applications by day 6 after CR infection - when Th17 differentiation is already initiated - did not alter the disease progression, and no differences in the colonic IL-17A expression nor in Th17 cell numbers were observed (Supplementary Fig. 2a–e), suggesting a negative contribution of IL-33 at the early phase of CR infection. Thus, we tested whether IL-33 may directly affect the ability of CD4^+^ T cells to differentiate into Th17 cells. Consistent with our in vivo findings, naïve CD4^+^ T cells showed a reduced capacity to differentiate into Th17 cells in the presence of IL33 in vitro (Fig. 6e). Importantly, the IL-17A expression per cell (MFI) was not changed (Fig. 6f), indicating that IL-33 does not affect the functional ability of differentiated Th17 cells to produce IL-17A. Moreover, we observed neither differences in the frequency of Foxp3^+^CD4^+^ T cells due to the presence of IL-33 (Fig. 6g) - thus excluding the possibility that IL-33 could promote Tregs differentiation at the expense of Th17 generation - nor alterations in the proliferative capacity of Th17-polarized CD4^+^ T cells (Fig. 6h). Together, these data suggest that IL-33 directly interferes with the generation of Th17 cells early during CR infection, which correlates with enhanced bacterial burden and severe pathology at later stage.

### IL-17A supplementation counterbalances the negative effect of IL-33 during *C. rodentium* infection

**Fig. 7:**
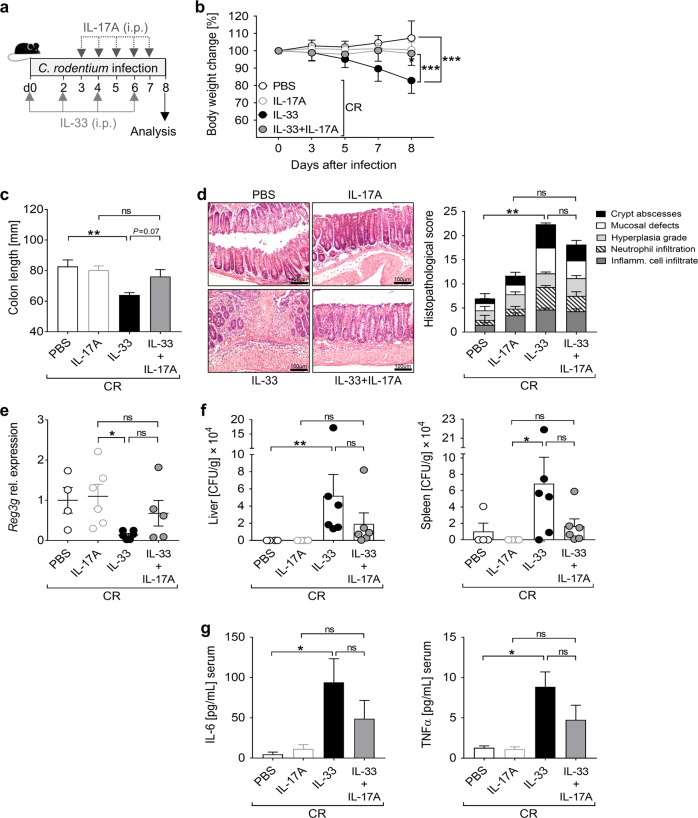
IL-17A supplementation during infection compensates the detrimental effect of IL-33. **a** CR-infected mice were treated with PBS (control group) or IL-33 as in Fig. 3a. Starting by day 3, one group of CR-infected IL-33-treated mice and one group of CR-infected non-treated mice received IL-17A daily until the end of the experiment. **b** Body weight changes during the course of infection are presented as percentage of initial weight on day 0. Statistical analyses were performed by two-way ANOVA followed by Bonferroni’s post hoc test. **c** Macroscopic score of colitis was assessed based on shortening of colon length. **d** Representative pictures (scale bars 100 µm) of H&E-stained colon sections (left) and histopathological score (right). **e** *Reg3g* mRNA expression in colon biopsies was determined by qRT-PCR and shown as fold change induction over PBS-treated group. **f** Systemic bacterial distribution upon IL-33 and/or IL-17A treatment was assessed on day 8 after infection in homogenized livers and spleens. **g** Serum cytokine levels were measured via Luminex technology. All data are presented as mean ± SEM and were pooled from two independent experiments (4–6 mice per group). Statistical analyses were performed using one-way ANOVA followed by Tukey’s multiple comparison test or Kruskal-Wallis test. *P < 0.05; **P < 0.01; ***P < 0.001.

To address whether IL-17A controls IL33-mediated immunopathology in our model, we applied IL-17A to IL-33-treated CR-infected mice from day 3 post infection on and characterized disease progression (Fig. 7a). Remarkably, IL-17A supplementation prevented body weight loss and colon length shortening in IL-33-treated infected mice (Fig. 7b, c). Furthermore, while CR-infected mice treated with IL-33 alone exhibited severe colon tissue damage, concomitant administration of IL33 and IL-17A resulted in a moderate reduction of colonic immunopathology (Fig. 7d) and a slight upregulation in Reg3γ expression (Fig. 7e). Accordingly, systemic bacterial dissemination and serum pro-inflammatory cytokine levels were less pronounced in IL-33+IL-17A infected animals compared to IL-33 treated infected mice (Fig. 7f, g).

To address the role of IL-17A specifically at the site of infection, we generated a genetically modified CR strain expressing and secreting IL-17A. Importantly, both wild-type (here named CR/WT) and IL-17A-producing (CR/IL17) CR strains showed the same gut colonization ability (Supplementary Fig. 3a). Consistent with the data obtained from IL-17A systemic application, infection with CR/IL17 limited the body weight loss and the systemic bacterial dissemination consequent to IL-33 treatment (Supplementary Fig. 3b–d). However, the constant production of IL-17A by the infectious bacteria was not sufficient to significantly ameliorate colon pathology (Supplementary Fig. 3e).

### IL-33 impairs gut barrier integrity by directly targeting IECs

**Fig. 8:**
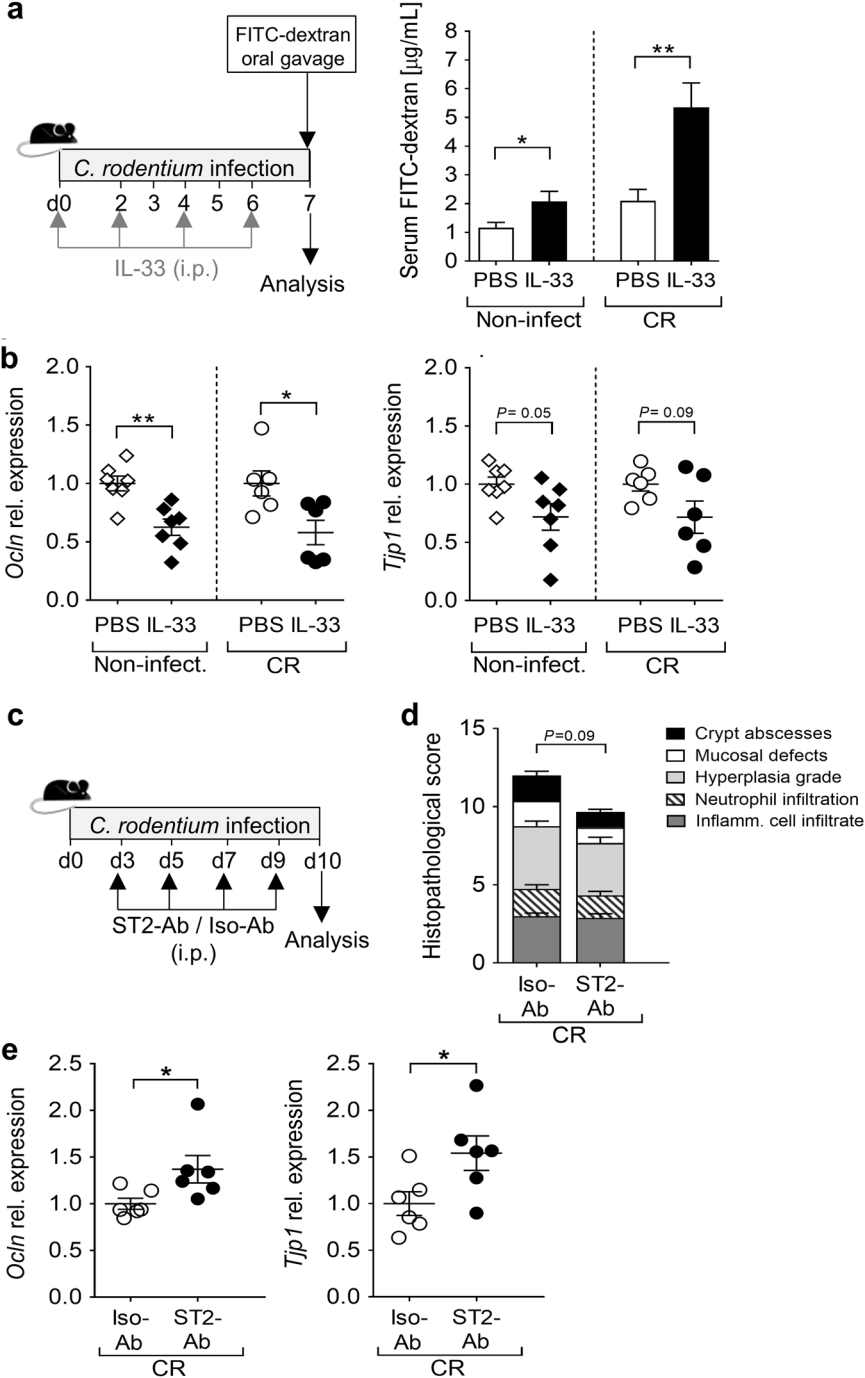
IL-33 impairs intestinal epithelial barrier integrity. **a** Mice were treated with IL-33 or PBS every 2 days for 1 week in presence or absence of concomitant CR infection. On day 7, mice received FITC-dextran beads via oral gavage. Serum FITC concentrations were measured 4h after gavage. Bars indicate the mean±SEM of FITC-dextran micrograms per milliliters of serum. **b** Colonic mRNA expression of the tight junction-related proteins *Ocln* (left) and *Tjp1* (right) determined via qRT-PCR. Graphs show dot plots and mean±SEM of fold change induction over PBS-treated groups. Results from three (non-infected, *n* = 10 mice per group) and two (CR-infected, *n* = 6 mice per group) independent experiments are shown. Statistical analyses were performed using Mann–Whitney U test or Student´s *t* test to compare IL-33-treated groups with their respective PBS-treated groups. **c** C57BL/6 mice were infected with CR and were treated with ST2-blocking antibody (ST2-Ab) or isotype antibody (Iso-Ab, control group) starting by day 3 after infection. **d** Histopathological analysis of colonic tissues based on the scoring system described in “Methods”. **e** Colonic mRNA expression of *Ocln* and *Tjp1* was assessed via qRT-PCR. Dot plots indicate fold change induction over Iso-Ab treated group. Statistical analysis was performed using Student’s *t* test. All data are presented as mean±SEM and were pooled from two independent experiments (*n* = 6 mice per group). Statistical analysis was performed using Mann–Whitney U test or Student’s *t* test to compare the ST2-Ab-treated group with the Iso-Ab control group. *P < 0.05; **P < 0.01; ***P < 0.001.

Given the substantial systemic bacterial dissemination found in IL-33-treated CR-infected animals, we tested whether IL-33 levels affected the integrity of the epithelial barrier in our model. First, both non-infected and CR-infected mice were treated with IL-33, and then orally gavaged with FITC-labeled dextran beads. Interestingly, IL-33 treatment resulted in significantly higher FITC concentrations in the serum, indicating enhanced gut permeability, an effect that was strengthened when IL-33 was applied during infection (Fig. 8a). Moreover, IL-33-treated animals showed substantial defects in the expression of occludin (Ocln) and, to a lesser extent, of tight junction protein 1 (Tjp1) within the colon (Fig. 8b). Well in line, the genetic ablation of IL-33 during CR infection resulted in enhanced expression of tight junction-related proteins (Supplementary Fig. 4a). Thus, we next analyzed whether an active inhibition of endogenous IL-33 signaling during CR infection using a ST2 blocking antibody (ST2-Ab) confers protection to the intestinal epithelium (Fig. 8c). Similar to our findings in *St2*^*−/−*^ mice, ST2-Ab treated CR-infected mice exhibited moderate less body weight loss and colon damage compared to infected animals receiving the isotype control (Fig. 8d and Supplementary Fig. 5). More importantly, colonic expression of both Ocln and Tjp1 was significantly higher in these mice (Fig. 8e), pointing out the detrimental role of IL-33/ST2 signaling on gut epithelium integrity during CR infection. To verify that IL-33 directly targets IECs and does not exert its effects indirectly via other immune cells or cytokines, we made use of murine intestinal epithelial MODE-K cell lines, which upregulate ST2 expression upon IL-33 treatment (Supplementary Fig. 4b). Notably, transcript levels of both *Ocln* and *Tjp1* were significantly reduced when cells were treated with IL-33 (Supplementary Fig. 4c), thus indicating a direct effect of IL-33 on gut epithelial cells.

### IL-17A counteracts the negative effect of IL-33 on gut permeability

**Fig. 9:**
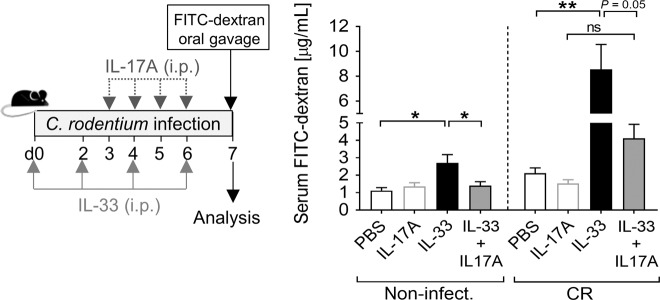
IL-17A counteracts the enhanced gut permeability induced by IL-33. Naïve and CR-infected mice were treated with IL-33 or PBS on day 0, 2, 4, and 6. Starting by day 3, one group of IL-33-treated mice and one group of non-treated mice received IL-17A daily. On day 7, serum FITC concentrations were measured after FITC-dextran oral gavage. Bars represent the mean ± SEM of FITC-dextran micrograms per milliliters of serum. Statistical analysis was performed using one-way ANOVA followed by Tukey’s multiple comparison test (non-infected, *n* = 4 mice per group, CR-infected, *n* = 6-7 mice per group). *P < 0.05; **P < 0.01; ***P < 0.001.

Inhibition of IL-17A signaling has been associated with impairment of the intestinal epithelial barrier and exacerbated colitis^21^. As we observed a negative effect of IL-33 on the gut barrier integrity during CR infection and that IL-17A supplementation limited the otherwise strong systemic spreading of the pathogen induced by IL-33, we combined the treatment with both cytokines in vivo under homeostasis and during CR infection, and assessed the intestinal permeability. Remarkably, IL-17A supplementation attenuated the enhanced gut permeability induced by IL-33 (Fig. 9).

We therefore conclude that IL-33 application impairs the integrity of the gut epithelium, which enhances the bacterial translocation from the intestinal lumen to systemic sites. Concomitantly, IL-33 inhibits the differentiation of IL-17A-producing CD4^+^ T cells, which have the potential to reverse the negative effect of IL-33 during infection. Taken together, our findings demonstrate that the balance between IL-17A and IL-33 levels is critical for the control of CR-mediated intestinal immunopathology.

## Discussion

The intestinal epithelium represents one of the first lines of defense against invading pathogens^22^. Alteration in the epithelium structure and barrier integrity are detrimental to the organism. CR is a non-invasive pathogen, and the infection is normally limited to the distal part of the colon^17^. Importantly, we observed a severe bacterial dissemination into the peripheral system with enhanced systemic inflammatory cytokine production in mice infected with CR and concomitantly treated with IL-33. Well in line with a study by Sedhom and colleagues^23^, our results demonstrated that IL-33 directly inhibits the expression of tight junction-related proteins in vitro and in vivo. Furthermore, IL-33 treatment strongly enhanced the intestinal permeability in vivo, thereby favoring bacterial translocation that amplifies systemic and colonic inflammation. Following CR infection, the upregulation of the antimicrobial peptide REG3γ in the colon was impaired by concomitant IL-33 application. Consequently, IL-33-treated CR-infected animals displayed elevated intestinal CR burdens. Xiao and colleagues recently showed that the steady-state expression of REG3γ in IECs of *Il33*^*−/−*^ mice is lower than in WT mice^24^. Of note, IL-33-deficient mice have been reported to be dysbiotic, or have a higher concentration of pro-inflammatory bacteria compared to WT mice^25^. Thus, the basal expression of REG3γ in *Il33*^*−/−*^ mice may be altered due to the complex interaction between intestinal epithelium, immune system and microbiota. Intriguingly, the authors showed that the in vitro treatment of intestinal epithelial cell lines with IL-33 induced the expression of REG3γ^24^. Yet it is not clear how these data compare with our, as the authors focused on IL-33 and REG3γ at steady-state, in cell culture conditions, and at the late phase of CR infection when the bacteria are almost cleared in WT mice. In our study, we concentrated on pathological aspects of acute CR infection within the colon of mice with impaired IL-33/ST2 signaling. In addition, we addressed the effect of exogenous IL-33 application on REG3γ expression in vivo during acute CR infection.

The balance between Tregs and Th17 cells is an important regulator of inflammation within the gut^26^. Interestingly, IL-33 is suggested to influence both Tregs and Th17 cells. ST2 is highly expressed on colonic Tregs at steady-state, and IL-33 was shown to increase their expression of ST2 and Foxp3 as well as to promote their suppressive function^27^. During CR-mediated colitis, Tregs protect the host against excessive immunity, but may also increase the risk of pathogen persistence and chronic disease. Wang et al. demonstrated that depletion of Tregs during the course of CR infection impairs bacterial clearance and the induction of a protective Th17 response^28^. In our model, IL-33 treatment of infected mice strongly increased the frequency of colonic Tregs; however, excessive inflammation in the colon was detected. In line with this finding, the depletion of Foxp3^+^Tregs in CR-infected mice treated with IL-33 did not rescue the phenotype and ameliorate the pathology, suggesting that the deleterious effects elicited by IL-33 are not dependent on the expansion of Tregs in our model. However, we cannot exclude that the expansion of ST2^+^Foxp3^+^ Tregs has some benefits for the host during CR infection, as mice with Treg-specific ST2 depletion showed slightly increased colon pathology compared to animals with ST2-sufficient Tregs.

CR infection elicits a strong Th17 response in the colon^29^. Through the production of IL-22 and IL-17, Th17 cells provide signals for the secretion of antimicrobial peptides within the intestinal epithelium^30^. Consequently, mice lacking IL-17A showed an impaired ß-defensin production in infected colons accompanied by increased CR burden^20^. Remarkably, our results show that IL-33 treatment during CR infection reduced the frequencies of IL-17A-expressing CD4^+^ T cells within the infected colon, and the impaired IL-17A response correlated with the uncontrolled pathogen clearance. Moreover, IL-33 directly inhibited the differentiation of Th17 cells in vitro. As ST2 expression by Th17 cells is very low at baseline, IL-33 should not impair the production of IL-17A in already in vitro polarized Th17 cells. This may explain why IL-33 treatment in the later phase of infection, when the differentiation of Th17 cells already has taken place, does not appear to affect CR-mediated immunopathology. Our findings raise the question whether the host may have a benefit from IL-33/ST2 deficiency or whether local IL-17A supplementation boosts host defense mechanisms against extracellular bacteria. Indeed, we observed that the re-establishment of IL-17A levels counteracts the strong systemic microbial dissemination elicited by IL-33 treatment during CR infection. IL-17A was suggested to be involved in preserving gut barrier functions. Lee et al. demonstrated that the genetic ablation or antibody neutralization of IL-17A signaling increased gut permeability in both chemically- and T cell transfer-induced colitis^31^. Moreover, IL-17A and IL-17RA inhibition have been associated with the breakdown of the intestinal epithelial barrier and exacerbated colitis in *Helicobacter bilis*-infected mice^21^. Here, we demonstrated that exogenous IL-17A supplementation abrogates the negative effect of IL-33 on the intestinal permeability. Nevertheless, administration of IL-17A during CR infection was not sufficient to rescue the severe immunopathology. This could be due to the fact that Th17 cells not only produce IL-17A but also IL-22^3^. In acute colonic infections, such as CR, where rapid repair of colonic epithelial cells is required, IL-22 plays a protective role by facilitating epithelium restoration and by enhancing IEC-derived antimicrobial peptides^32^. Interestingly, supplementation of IL-33-treated CR-infected mice with exogenous IL-22 neither ameliorated colon pathology nor rescued the downregulation of *Reg3g* expression induced by IL-33 treatment (data not shown). Thus, an impaired IL-22 signaling does not account for the pathogenic potential of IL-33 in our infectious model.

IL-33 is frequently described to promote type-2 immunity by inducing ILC2s and Th2 cells^5,8^, which could inhibit an effective Th1/Th17 response. Notably, colonic type-2 cytokine levels and the frequency of ILC2 cells were increased in IL-33 treated non-infected mice (Supplementary Fig. 6a, b). However, in IL-33 treated CR-infected mice these enhanced type-2 responses were almost abolished, probably due to a counteracting Th1 cytokine response and higher frequencies of IL-17 producing ILC3 cells induced by CR^33^. Nonetheless, IL-33 treatment during CR infection induced a low percentage of ILC2 to the detriment of ILC3 (Supplementary Fig. 6b), which may affect the subsequent development of an efficient adaptive immune response. Thus, we cannot completely exclude that a misbalance between ILC2s and ILC3s might also contribute to the reduced Th17 response during CR infection.

In the present study, we found that IL-33 levels in the colon were only slightly increased following CR infection, but the expression of the IL-33-receptor ST2 was significantly enhanced in the colon of CR*-*infected mice. Similar expression profiles were observed in patients suffering from enterohemorrhagic *E. coli* (EHEC)-driven hemolytic uremic syndrome (HUS)^34^. Whereas IL-33 serum levels in HUS patients were not altered compared to healthy controls, the serum levels of sST2 were significantly increased in the HUS phase and correlated with disease severity^34^. However, the functional impact of IL-33/ST2 pathway on EHEC infections is still unrevealed.

IL-33 and IL-17A have been separately described to play dichotomous functions in the gut depending on the infection context. Colonization with the pathobiont adherent-invasive *E. coli* (AIEC) was shown to potentiate the expression of IL-33 and ST2 in the intestine mediated by the bacterial flagellin^35^. Furthermore, AIEC colonization of *Il17*^−/−^ mice resulted in increased intestinal epithelial damage, systemic bacterial dissemination and mortality, indicating that IL-17 plays a protective role in AIEC-induced colitis^36^. Thus, we suggest that the flagellin-driven expansion of ST2 and IL-33 could be a self-protective mechanism of AIEC to counterbalance the induction of Th17-mediated responses.

In contrast, IL-33 signaling was described to be critical for intestinal protection during *Clostridium (C.) difficile* colitis. Frisbee and colleagues demonstrated that colonic IL-33 was amplified in response to mouse and human *C. difficile* infection, and a dysregulation of IL-33 signaling was associated with higher patient mortality^37^. Interestingly, in humans, Th17 cytokines in the serum are associated with severe *C. difficile* infection, and adoptive transfer of Th17 cells to *C. difficile-*infected mice increased mortality^38^. In this setting, enhanced colonic IL-33 expression during *C. difficile* infection could protect the host by inhibiting the differentiation of Th17 cells. Taken together, these and our data indicate that IL-33 and IL-17A adversely regulate the host response during infection with different intestinal bacteria.

Based on our results, it is tempting to speculate about a temporal hierarchy of IL-33/IL-17A effects in the host response to gut pathogens. IL-33 is highly abundant in the intestine at steady-state, and the expression of IL-33 or ST2 enhances upon tissue damage. In contrast, Th17 cells belong to the adaptive immune response that starts in a later phase of infection. Since IL-33 treatment does not impair the immune response in the late phase of CR infection, it is likely that IL-17A has the power to counterbalance the early pathogenic potential of IL-33 within the gut. However, additional experiments are needed to better characterize the reciprocal regulation of intestinal IL-33 and IL-17A levels during the course of bacterial infection.

In summary, our findings extend the broad spectrum of functions of IL-33, and highlight the special role of the gut as an organ with systemic immune-regulatory properties. Exploring the crucial and diverse roles of the IL-33/ST2 axis during infections may help in the development of therapeutic interventions for a range of intestinal infectious diseases.

## Methods

### Mice

All animal experiments at the University Hospital Essen were performed in accordance with ethical principles and federal guidelines, and approved by the local authority Landesamt für Natur, Umwelt und Verbraucherschutz (LANUV, North-Rhine Westphalia, Germany). C57BL/6 J WT mice (6–8 week old females) were purchased from Envigo (Horst, Netherlands). DEREG transgenic mice were established as previously described^39^, and bred in-house. ST2^fl/fl^/FIC mice were generated by crossing *Il1rl1*^tm1c(KOMP)Wtsi^ (www.komp.org)^40^ with Foxp3^tm1(cre)Saka^ mice^41^. All animal experiments at the University of Bern were performed in agreement with Swiss Federal regulations and were approved by the Cantonal Veterinary Office of Bern, Switzerland. Il1rl1^tm1Anjm^ (*St2*^*−/−*^) mice were previously described^42^, and backcrossed on a C57BL/6 J background. *Il33*^*−/−*^ mice were obtained through the RIKEN Center for Developmental Biology (http://www.cdb.riken.jp/arg/mutant%20mice%20list.html)^43^, and bred in-house. For all experiments, mice were either co-housed (for females), or soiled bedding was exchanged weekly (for males).

### Bacterial strains and infection of mice

Bacterial-driven colitis was induced in mice by oral inoculation with ~2 × 10^9^ colony forming units (CFU) of WT CR strain ICC169, as previously described^44^. In some studies, a genetically modified CR strain was used, which constitutively secretes IL-17A (named CR/IL-17) (see Supplementary methods for construct details).

### In vivo treatments

Recombinant mIL-33 (1 µg/mouse, BioLegend) was administered i.p. to the mice immediately upon infection on day 0 and every two days for the duration of the experiment. Where indicated, recombinant mIL-17A (1 µg/mouse, BioLegend) was supplemented i.p. daily, starting by day 3 after infection. As late treatment, IL-33 was administered every two days starting by day 6 after infection. For pharmacological inhibition of endogenous IL-33/ST2 signaling, a blocking antibody against ST2 (20 µg/mouse, R&D Systems) was supplemented i.p. every two days starting by day 3 post infection.

### Histopathological analysis

Full-length colons were stored in 4% paraformaldehyde and embedded in paraffin. Sections (4 μm) were prepared from paraffin-embedded blocks, stained with hematoxylin and eosin (H&E) and evaluated in a blinded manner, according to standard techniques as described previously^45^. In brief, middle and distal colon portions were assessed for damage and inflammation (each parameter analyzed was scored 0 = none; 1 = mild; 2 = moderate; 3 = severe change).

### Protein quantification

sST2 concentrations in serum were measured using a commercially available enzyme-linked immunosorbent assay (ELISA) kit for murine sST2 (ThermoFisher). Cytokine levels in the serum were quantified using polystyrene bead-based Luminex technology (R&D Systems). Quantification of cytokines secreted in the supernatants of 6-hour in vitro cultured colonic explants was performed as previously described^46^, using a Luminex 200 system (Luminex Corporation).

### Isolation of colonic lamina propria lymphocytes

Lamina propria lymphocytes (LPLs) were isolated from the colon as described in Supplementary methods.

### In vitro Th17 cell differentiation and proliferation assay

Naïve CD4^+^ T cells were isolated from the spleens of C57BL/6 mice using the CD4^+^ T Cell Isolation Kit II (Miltenyi Biotec). CD4^+^ CD25^-^ T cells were sorted using a FACSAria II cell sorter (BD Biosciences). Purified cells were cultured for 5 days with plate-bound anti-CD3 (clone 145–2C11) (1 μg/mL) and soluble anti-CD28 (clone 37.51) (1 μg/mL) (both BD Bioscience). To induce Th17 differentiation, culture medium was supplemented with recombinant TGFβ (2 ng/mL), IL-21 (100 ng/mL), IL-23 (20 ng/mL), IL-6 (50 ng/mL), IL-1β (20 ng/mL), and blocking antibodies for IL-4 (200 ng/mL), IFN-γ (200 ng/mL), and IL-2 (200 ng/mL), respectively. Where indicated, rmIL-33 (10 ng/mL) (BioLegend) was added to the culture. On day 5, cells were counted, stained and analyzed by flow cytometry as described below. To assess cell proliferation, sorted-purified CD4^+^ CD25^-^ T cells were labeled with the cell proliferation dye eFluor 670 (eBioscience) following the manufacturer’s instructions prior to 5-day stimulation with or without IL-33 under Th17 cell-skewing conditions. Loss of eFluor 670 was assessed by flow cytometry as indicative of cell proliferation.

### Antibodies and flow cytometry

Cells were incubated with marker-specific fluorochrome-labeled anti-mouse antibodies listed in Supplementary Table 2 and described in Supplementary methods. Cells were analyzed by flow cytometry on a LSR II instrument using DIVA software (BD Biosciences).

### FITC-dextran intestinal permeability assay

Mice were orally gavaged with 150 μl of 100 mg/mL 4 kDa FITC-dextran-labeled dextran beads (Sigma-Aldrich) in PBS 4 h prior to sacrifice. FITC-derived fluorescence was quantified in the serum using a microplate luminometer (Mithras LB 943; Berthold). Concentrations were determined using a standard curve generated by serial dilution of FITC-dextran.

### RNA extraction and quantitative RT-PCR

RNA was isolated from colon biopsies as described before^47^. Primer sequences are listed in Supplementary Table 1. Relative mRNA levels were determined based on the standard curves for each individual gene and normalized to the expression of the housekeeping gene ribosomal protein S9 (RPS9).

### Statistical analysis

All analyses were performed using the Prism 7.03 software (GraphPad, La Jolla, CA). Unless specified, only statistically significant differences are indicated in the figures. *P < 0.05; **P < 0.01; ***P < 0.001.

## Supplementary information

Supplementary methods
