# Supplementary material for: Interleukin-33 signaling exacerbates experimental infectious colitis by enhancing gut permeability and inhibiting protective Th17 immunity
Source: Mucosal Immunol. 2021 Mar 2;14(4):923–36. doi: 10.1038/s41385-021-00386-7 (PMC8221996; doi:10.1038/s41385-021-00386-7)
Supplement: Supplementary file 1 — Supplementary methods [file 41385_2021_386_MOESM1_ESM.pdf]

Interleukin-33 signaling exacerbates experimental infectious colitis by enhancing gut permeability and inhibiting protective Th17 immunity

Vittoria Palmieri[1], Jana-Fabienne Ebel[1], Nhi Ngo Thi Phuong[1], Robert Klopfleisch[2], Vivian Pham Vu[3,4], Alexandra Adamczyk[1], Julia Zöller[1], Christian Riedel[5], Jan Buer[1], Philippe Krebs[3], Wiebke Hansen[1], Eva Pastille[1], Astrid M. Westendorf[1*]

[1] Institute of Medical Microbiology, University Hospital Essen, University of Duisburg-Essen, Essen, Germany

[2] Institute of Veterinary Pathology, Free University of Berlin, Berlin, Germany

[3] Institute of Pathology, University of Bern, Bern, Switzerland

[4] Graduate School for Cellular and Biomedical Sciences, University of Bern, Bern, Switzerland

[5] Institute of Microbiology and Biotechnology, University of Ulm, Ulm, Germany

Supplementary Methods

Generation of IL-17-producing *C. rodentium* strain.

In order to obtain an engineered *C. rodentium* strain that express functional IL-17A, a synthetic DNA construct comprising the P_{help} promoter (Riedel et al., 2007) and the signal peptide of the appA gene of *Escherichia coli* K-12 fused to the coding sequence for murine IL-17 amino acid residues 26-158 (i.e. the protein without its native signal peptide) was designed. The construct was codon-optimized for expression in *C. rodentium* and amplified by PCR using primers SacII_Phelp_fwd and IL17_KpnI_rev. This plasmid was introduced into CR ICC169: p16S_PT5mRuby (Manta et al., 2013) and confirmed by sequencing and restriction analysis.

References:

Riedel C.U., Monk I.R., Casey P.G., Morrissey D., O'Sullivan G.C., Tangney M. *et al.* Improved luciferase tagging system for Listeria monocytogenes allows real-time monitoring in vivo and in vitro. *Appl Environ Microbiol* **73**, 3091-3094 (2007).

Manta C., Heupel E., Radulovic K., Rossini V., Garbi N., Riedel C.U. *et al.* CX(3)CR1(+) macrophages support IL-22 production by innate lymphoid cells during infection with Citrobacter rodentium. *Mucosal Immunol* **6**, 177-188 (2013).

Isolation of colonic lamina propria lymphocytes.

Lamina propria lymphocytes (LPLs) were isolated from the colon. Briefly, colons were opened longitudinally and cut into 1-cm pieces. Tissue pieces were washed in PBS containing 3mM Ethylene-diamine-tetra-acetic acid (EDTA) for 10 min at 37°C with shaking. EDTA was removed by washing colon pieces twice in Roswell Park Memorial Institute (RPMI-1640) medium containing 1% fetal calf serum (FCS), 1mM EGTA, and 1.5mM $MgCl_2$ for 15 min at 37°C with shaking. After colon pieces were accurately minced, single cells were obtained by further digestion in RPMI containing 20% FCS and 100U/mL collagenase IV (Clostridium histolyticum; Sigma-Aldrich, St. Louis, MO) for 60 min at 37°C, followed by filtration through 40μm cell strainer. Single cells were finally suspended in culture medium and processed for flow cytometry analysis.

Antibodies and flow cytometry.

Cells were incubated with marker-specific fluorochrome-labeled anti-mouse antibodies listed in Supplementary Table 2. For analysis of intracellular IL-17A, isolated LPLs or *in vitro*-polarized $CD4^+$ T cells were stimulated for 4 hours with 10 ng/mL phorbol 12-myristate 13-acetate (PMA) and 1μg/mL ionomycin in the presence of 5 μg/mL Brefeldin A (all Sigma-Aldrich) in complete medium. After surface staining, intracellular detection of Foxp3 and IL-17A was performed using the Foxp3 fixation/permeabilization kit (eBioscience). Cells were analyzed by flow cytometry on a LSR II instrument using DIVA software (BD Biosciences).

Intestinal epithelial cell culture.

MODE-K cells were maintained in Dulbecco's Modified Eagle's Medium, supplemented with 2mM L-glutamine, 1% PenStrep, and 10% FCS in a humidified atmosphere at 37°C. Cells were seeded on 24-well plates and incubated overnight. The following day, medium was replaced by fresh medium supplemented or not with recombinant mIL-33 (10ng/mL), and cells were incubated for additional 6 hours. Cells were harvested for RNA extraction and qRT-PCR analysis.

Supplementary Figures

Supplementary Fig. 1

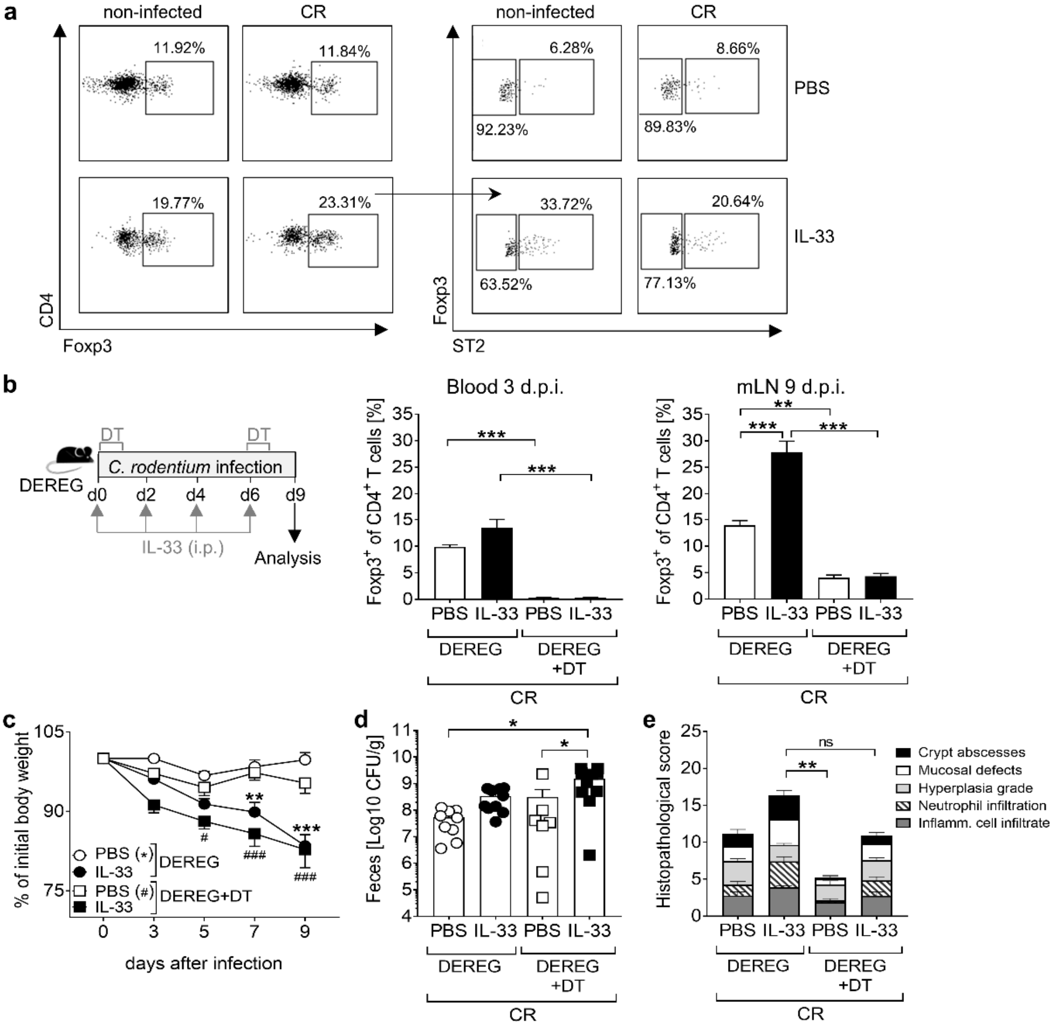

Supplementary Fig. 1. Treg depletion during CR infection is not sufficient to rescue the detrimental effect of IL-33 on colitis. **a.** Representative flow cytometry dot plots showing the frequency of Foxp3$^+$ Treg cells gated on CD4$^+$ T cells and of ST2$^+$ cells among Tregs in the colon of non-infected and CR-infected mice treated with PBS or IL-33 related to Fig. 5a. Results are shown as the mean of Foxp3$^+$ Treg or ST2$^+$ Treg cell frequency for each experimental group. **b.** Schematic illustration of IL-33 treatment during infection in DEREG transgenic mice. To deplete Treg cells during infection, two cycles of diphtheria toxin administration were performed (DT groups). Treg ablation was confirmed in blood and mesenteric lymph nodes (mLN) of DT-treated mice, respectively on day 3 and 9 post infection (d.p.i.) by flow cytometry. Bars represent the mean ± SEM (n = 3-8 mice per group). Statistical analyses were performed using one-way ANOVA followed by Tukey's multiple comparison test. **c.** Body weight changes monitored along the course of infection. Statistics were obtained using two-way ANOVA followed by Bonferroni's post hoc test. IL-33- vs PBS-treated DEREG mice *$P < 0.05$; **$P < 0.01$; ***$P < 0.001$. IL-33- vs PBS-treated DEREG+DT mice $^{\#}P < 0.05$; $^{\#\#}P < 0.01$; $^{\#\#\#}P < 0.001$ **d.** Intestinal bacterial load was assessed by plating serial dilutions of fecal samples on MacConkey agar at the end of the experiment. **e.** Histopathological score of colon tissues. All data are presented as mean ± SEM. Results from three independent experiments are shown. Statistical analyses were performed using one-way ANOVA followed by Tukey's multiple comparison test or Kruskal-Wallis test. *$P < 0.05$; **$P < 0.01$; ***$P < 0.001$

Supplementary Fig. 2

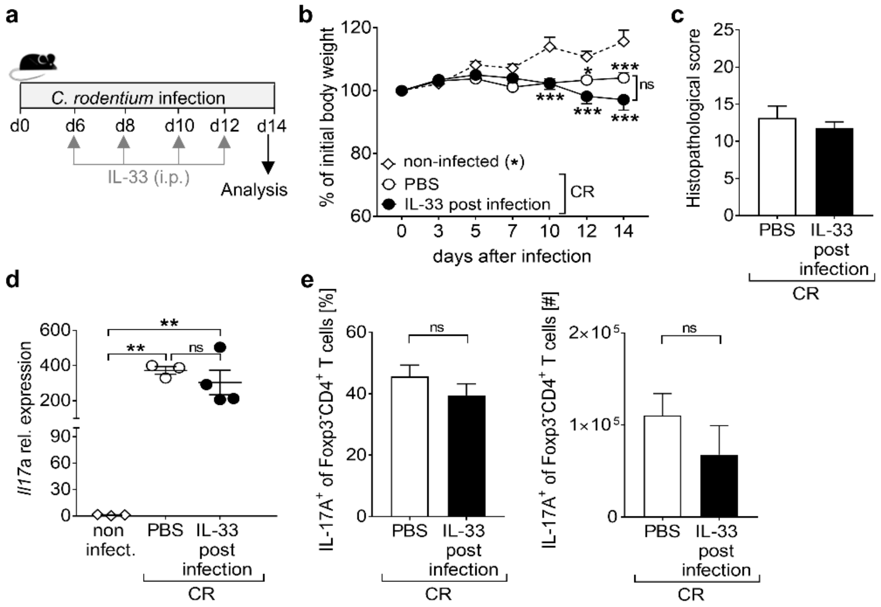

Supplementary Fig. 2. Administration of IL-33 in the late phase of *C. rodentium* infection has no impact on bacterial-induced colitis. **a.** C57BL/6 mice were infected with CR on day 0. By day 6 after infection, mice were treated with IL-33 or PBS (control group) every two days and sacrificed on day 14 post infection. **b.** Changes in body weight were assessed as percentage of initial weight on day 0. Statistical analyses were performed using two-way ANOVA followed by Bonferroni's post hoc test. **c.** Histopathological analysis of H&E-stained colon sections based on the scoring system described in Methods. Bars show the mean ± SEM of 3-4 mice per group. Mann-Whitney U test was used for statistical analysis. **d.** qRT-PCR analysis of *Il17a* expression in colon biopsies 14 days after infection. Data indicate fold change induction over non-infected group. Statistical analysis was performed using one-way ANOVA followed by Tukey's multiple comparison test. **e.** Colonic frequencies (left) and absolute numbers (right) of IL-17A-expressing cells among Foxp3$^-$CD4$^+$ T cells (Th17) were determined by flow cytometry (n = 3-4 per group). Bars indicate the mean ± SEM. Mann-Whitney U test was used for statistical analysis. *P < 0.05; **P < 0.01; ***P < 0.001

Supplementary Fig. 3

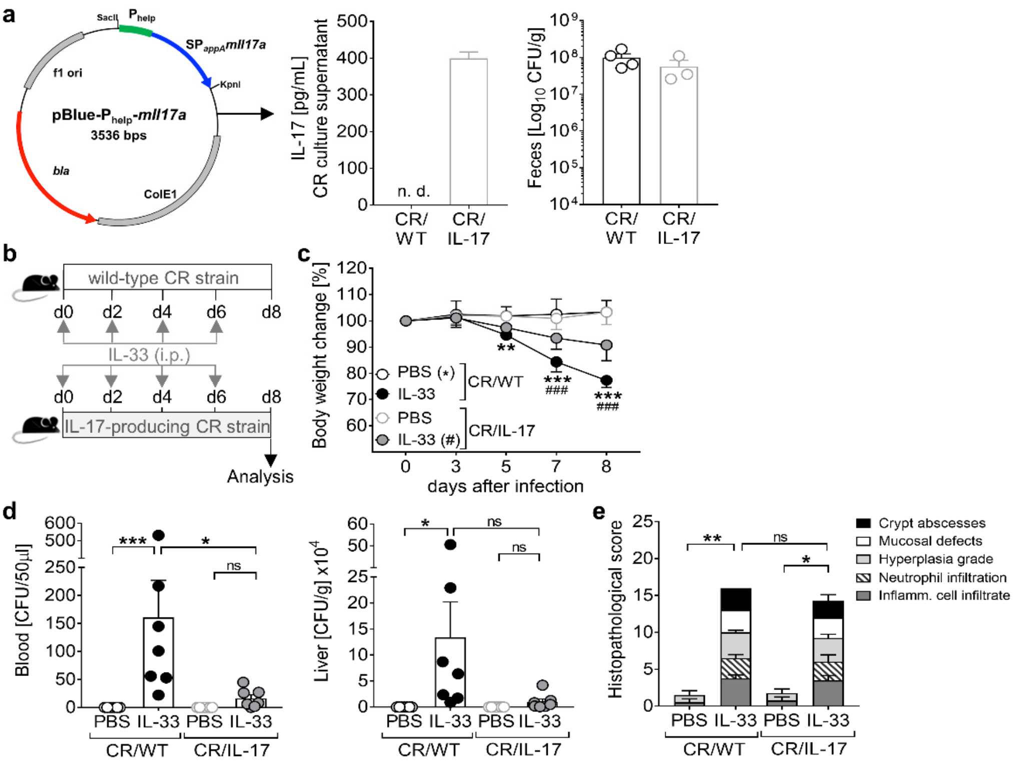

Supplementary Fig. 3. Local IL-17A production limits the systemic bacterial spread upon IL-33 treatment. **a.** Plasmid introduced into CR (CR/IL-17) for the expression and secretion of active IL-17A. IL-17A production was determined in the supernatants of O/N bacterial cultures from both the *C. rodentium* strains (CR/WT and CR/IL-17) via Luminex technology. Bars show the mean ± SEM of cytokine picograms per milliliters of bacterial culture supernatant. Effectiveness of bacterial colonization *in vivo* determined by plating feces from CR/IL17-infected and CR/WT-infected mice on MacConkey agar. **b.** Schematic illustration of IL-33 application during infection with the two different CR strains. On day 0, two groups of mice were orally gavaged with CR/IL17 and two other groups were infected with the respective wild-type bacterial strain as controls (CR/WT). Each group received either PBS or IL-33 intraperitoneally on day 0, 2, 4 and 6 after infection. **c.** Body weight changes were monitored along the course of infection. Statistical analyses were performed by two-way ANOVA followed by Bonferroni's post hoc test. The graph shows data from two independent experiments (7 mice per group). **d.** Systemic bacterial distribution after infection was assessed by plating whole blood (left) and serial dilutions of homogenized livers (right) on MacConkey agar. **e.** Histopathological score of H&E-stained colon tissue sections. Bars show the mean ± SEM of each inflammatory parameter. Data from one representative experiment are shown (n = 4 mice per group). Statistical analyses were performed using one-way ANOVA followed by Tukey's multiple comparison test. $^*P < 0.05$; $^{**}P < 0.01$; $^{***}P < 0.001$. $^{\#}P < 0.05$; $^{\#\#}P < 0.01$; $^{\#\#\#}P < 0.001$.

Supplementary Fig. 4

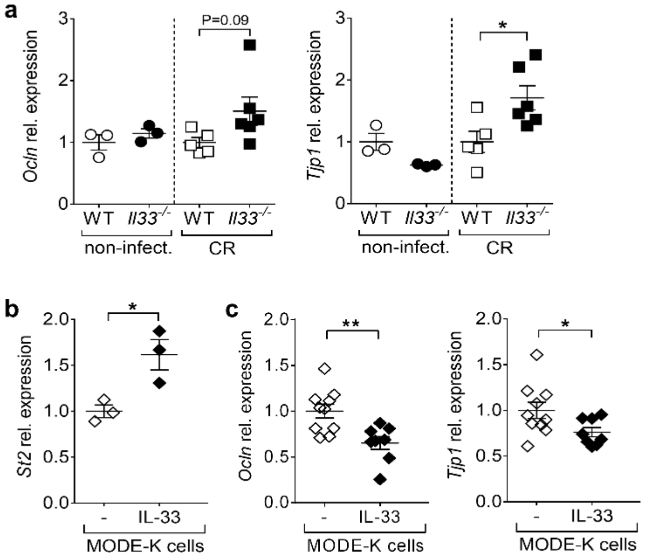

Supplementary Fig. 4. Effect of IL-33 on IECs. **a.** qRT-PCR analysis of Ocln and Tjp1 expression in colon biopsies of non-infected and CR-infected WT and *Il33*$^{-/-}$ mice. Data from one experiment are shown as fold change induction over the WT group. Statistical analysis was performed using Student's t-test to compare *Il33*$^{-/-}$ groups with their respective WT control groups. **b,c.** Confluent MODE-K cells were cultured in presence or absence of IL-33 for 6 hours and processed for RNA extraction. Transcript levels of the membrane-bound receptor *St2* **(b)**, and *Ocln* and *Tjp1* **(c)** were measured via qRT-PCR. Dot plots and the mean ± SEM indicate fold change induction over non-treated cells. Results from **c** one and **d** three independent experiments are shown. Statistical significances were obtained using Paired t-test. *P < 0.05; **P < 0.01; ***P < 0.001.

Supplementary Fig. 5

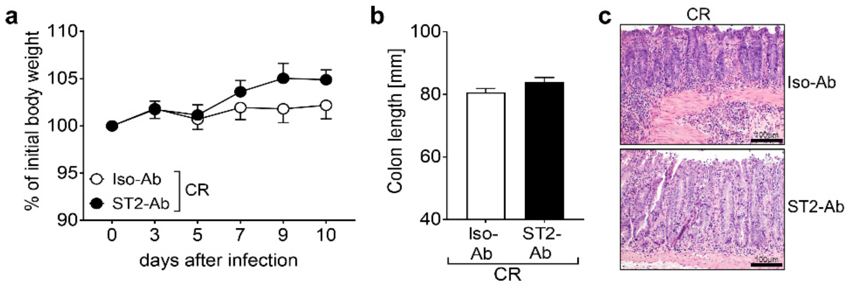

Supplementary Fig. 5. Effect of ST2 blockade during the course of CR infection. **a.** Body weight changes relative to initial weight were monitored in CR-infected mice treated with ST2-blocking antibody (ST2-Ab) or isotype antibody (Iso-Ab) as described in Fig. 8c. Statistical analyses were performed using two-way ANOVA followed by Bonferroni's post hoc test. Data from two independent experiments are shown (n = 6 mice per group). On day 10 post infection, colons were harvested for assessment of colitis. **b-c.** Colon length shortening and representative pictures (scale bars 100µm) of H&E-stained colon sections related to Fig. 8d.

Supplementary Fig. 6

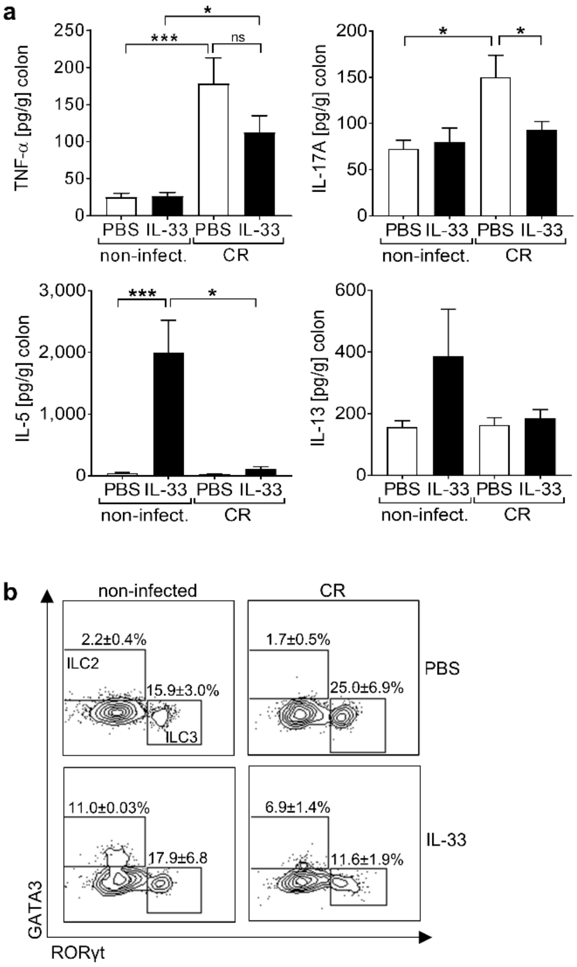

Supplementary Fig. 6. Cytokine release and proportion of ILC2 and ILC3 following CR infection and/or IL-33 challenge. **a.** Secretion of TNF-α, IL-17A, IL-5 and IL-13 in the supernatants of *in vitro* cultured colon biopsies from naïve and CR-infected mice that were treated with PBS or IL-33 as illustrated in Fig. 3a, determined by Luminex technology. Bars represent the mean ± SEM of data from four independent experiments (n = 9-15 mice per group). Statistical analyses were performed using one-way ANOVA followed by Tukey's multiple comparison test or Kruskal-Wallis ANOVA with Dunn's multiple comparison test. *$P < 0.05$; **$P < 0.01$; ***$P < 0.001$ **b.** Colonic frequency of ILC2 and ILC3. Mice were treated as described in Fig. 3a. Cells were isolated from the colonic lamina propria at day 8 after infection and processed for FACS analysis. Representative flow cytometry dot plots showing the gating strategy applied to assess the percentage of ILC2 (Lin^- $CD127^+GATA3^+$) and ILC3 (Lin^- $CD127^+ROR\gamma t^+$) subsets (n = 4 mice per group). Data are presented as mean ± SEM.

Supplementary Tables

Supplementary Table 1. List of anti-mouse antibodies used for flow cytometry.

Specificity	Conjugate	Clone	Manufacturer	Cat. number
CD4	APC	RM4-5	BD Biosciences	553051
CD4	Pacific Blue	RM4-5	BD Biosciences	558107
CD8	Brillian Violet 510	53-6.7	BioLegend	100752
CD8	Pacific Blue	53-6.7	BD Biosciences	558106
Foxp3	FITC	FJK-16s	eBioscience	11-5773-82
ST2	PE-Cy7	RMST2-2	eBioscience	25-9335-82
ST2	APC	RMST2-2	eBioscience	17-9335-82
IL-17A	PE	TC11-18H10.1	BD Biosciences	559502
CD45	PerCP-Cy5.5	30-F11	BioLegend	103131/103132
CD11c	PE	N418	BioLegend	117307/117308
F4/80	FITC	BM8	Invitrogen	11-4801-82
CD11b	APC	M1/70	eBioscience	17-0112
Ly6G	Brillian Violet 421	1A8	BD Biosciences	562737
CD3	FITC	17A2	BioLegend	100203
B220	FITC	RA3-6B2	BD	553087
CD19	FITC	1D3	BD	557398
CD11b	FITC	M1/70	ebioscience	53-0112-80
F4/80	FITC	BM8	eBioscience	12-4801-82
CD11c	FITC	HL3	BD	553801
NK1.1	FITC	PK136	BD Biosciences	553164
GATA3	PE	TWA5	eBioscience	12-9966-42
RORγt	APC	AFKJS-9	eBioscience	17-6988-80
CD127	eFluor450	A7R34	eBioscience	48-1271-82
Fixable viability dye	eFluor 780		eBioscience	65-0865-14

Supplementary Table 2. List of primer sequences used for qRT-PCR.

Gene	qPCR Antisense Primer (5' to 3')	qPCR Antisense Primer (5' to 3')
RPS9	CTGGACGAGGGCAAGATGAAGC	TGACGTTGGCGGATGAGCACA
St2	TGTGAGCCGTGTGAGTTTGAGTGT	TGGAGCAGCAGGCATGAGGAAGC
Il33	CTACTGCATGAGACTCCGTTCTG	AGAATCCCGTGGATAGGCAGAG
Il17a	GCTCCAGAAGGCCCTCAGACTACC	TTCCCTCCGCATTGACACAGC
Reg3g	GGCTCCATGACCCGACACT	TAGGCCTTGAATTTGCAGACATAG
Ocln	GCCCTGGCTGACCTAGAACTTAC	AGACTTAGCCAAAACTGCCTTAGC
Tjp1	TTTTTGACAGGGGGAGTGG	TGCTGCAGAGGTCAAAGTTCAAG
